# Sialoadhesin Expressed on IFN-Induced Monocytes Binds HIV-1 and Enhances Infectivity

**DOI:** 10.1371/journal.pone.0001967

**Published:** 2008-04-16

**Authors:** Hans Rempel, Cyrus Calosing, Bing Sun, Lynn Pulliam

**Affiliations:** 1 Department of Laboratory Medicine at the Veterans Affairs Medical Center, San Francisco, California, United States of America; 2 University of California San Francisco, San Francisco, California, United States of America; Beijing Institute of Infectious Diseases, China

## Abstract

**Background:**

HIV-1 infection dysregulates the immune system and alters gene expression in circulating monocytes. Differential gene expression analysis of CD14^+^ monocytes from subjects infected with HIV-1 revealed increased expression of sialoadhesin (Sn, CD169, Siglec 1), a cell adhesion molecule first described in a subset of macrophages activated in chronic inflammatory diseases.

**Methodology/Principal Findings:**

We analyzed sialoadhesin expression on CD14^+^ monocytes by flow cytometry and found significantly higher expression in subjects with elevated viral loads compared to subjects with undetectable viral loads. In cultured CD14^+^ monocytes isolated from healthy individuals, sialoadhesin expression was induced by interferon-α and interferon-γ but not tumor necrosis factor-α. Using a stringent binding assay, sialoadhesin-expressing monocytes adsorbed HIV-1 through interaction with the sialic acid residues on the viral envelope glycoprotein gp120. Furthermore, monocytes expressing sialoadhesin facilitated HIV-1 *trans* infection of permissive cells, which occurred in the absence of monocyte self-infection.

**Conclusions/Significance:**

Increased sialoadhesin expression on CD14^+^ monocytes occurred in response to HIV-1 infection with maximum expression associated with high viral load. We show that interferons induce sialoadhesin in primary CD14^+^ monocytes, which is consistent with an antiviral response during viremia. Our findings suggest that circulating sialoadhesin-expressing monocytes are capable of binding HIV-1 and effectively delivering virus to target cells thereby enhancing the distribution of HIV-1. Sialoadhesin could disseminate HIV-1 to viral reservoirs during monocyte immunosurveillance or migration to sites of inflammation and then facilitate HIV-1 infection of permissive cells.

## Introduction

Blood monocytes constitute an important immune cell population that is adversely impacted by HIV-1 infection. Monocytes originate in the bone marrow from myeloid precursors [1] and are released to circulation where their half-life in humans is about three days [2]. During their short life span, monocytes can differentiate to become either macrophages [3] with prolific degradative capacity [4] or dendritic cells (DCs), which effectively prime T cells by presenting antigens [5]. While macrophages and DCs are readily infected by R5 HIV-1 strains, monocytes are considered refractory to HIV-1 infection [6] with <1% of blood monocytes infected [7]. Even at this low rate of infection, HIV-1 appears to enter the central nervous system (CNS) via infiltrating monocytes [8], [9], which through the release of neurotoxins initiates the neurodegenerative processes that may end in HIV-associated dementia (HAD).

While the frequency of HAD has decreased with prevalent use of highly active antiretroviral therapy (HAART), neurocognitive impairment remains a reality in a substantial number of individuals infected with HIV-1. A link between viral load and impaired neural function is suggested by a recent study in non-human primates indicating that monocytes may be the link between HIV-1 in the periphery and HAD. Using an SIV-infected macaque model, Williams et al., reported that neuronal injury was coincident with viremia and an activated monocyte subset [10]. By lowering the systemic viral load with antiretroviral therapy, there was a commensurate reduction in the number of infected and activated monocytes and a dramatic improvement in neuronal function [10]. These observations establish a compelling link between high viral load, activated monocytes, an increased frequency of monocyte trafficking and a direct, negative impact on neuronal function.

To identify cellular factors that might contribute to HIV-1 invasion of the CNS, we examined gene expression profiles of CD14^+^ monocytes from individuals infected with HIV-1. Using high-density cDNA microarrays, we compared gene expression profiles from subjects with high viral load (>10,000 RNA copies/ml), subjects with low viral load (<10,000 RNA copies/ml) and HIV-1 seronegative controls [11]. We observed a monocyte gene expression profile related to HIV-1 infection that indicated a “hybrid” monocyte with increased expression of macrophage-associated markers: monocyte chemotactic protein-1 (MCP-1, CCL2), CC-chemokine receptor 5 (CCR5), and sialoadhesin (Sn, CD169, Siglec 1) [11]. This was the first report of Sn expression in circulating CD14^+^ monocytes.

Sialoadhesin was first described as a lymphocyte cell adhesion molecule expressed on macrophages localized in secondary lymphoid organs [12] and later as a protein restricted to a subset of activated macrophages related to inflammatory responses associated with rheumatoid arthritis and atherosclerosis [13]. More recently, Sn has been implicated in diverse pathogenic processes including rhinovirus infection [14] and porcine reproductive and respiratory syndrome virus infection [15]. In HIV infection, Sn is induced to high levels on CD14^+^ monocytes shortly after infection, possibly contributing to dysregulation of the immune system [16]. Sialoadhesin preferably binds Neu5Ac in α2,3 glycosidic linkage [17], [18] and as the largest of the Siglecs, Sn engages sialic acid conjugates on adjacent cells mediating cell-cell interactions [19]. In contrast, shorter Siglecs bind sialic acid conjugates in a *cis* orientation.

In this study, we report that HIV-1 infection drives monocyte expression of Sn, which correlates with viral load in the periphery. We identify interferons (IFN), which have been detected in the periphery of individuals infected with HIV-1, as inducers of Sn expression in cultured monocytes. Furthermore, using a constitutive Sn-expressing cell line and IFN-stimulated primary monocytes, we describe how Sn avidly binds HIV-1 and effectively facilitates *trans* infection of permissive cells.

## Results

### Sialoadhesin expression correlates with viral load

**Figure 1:**
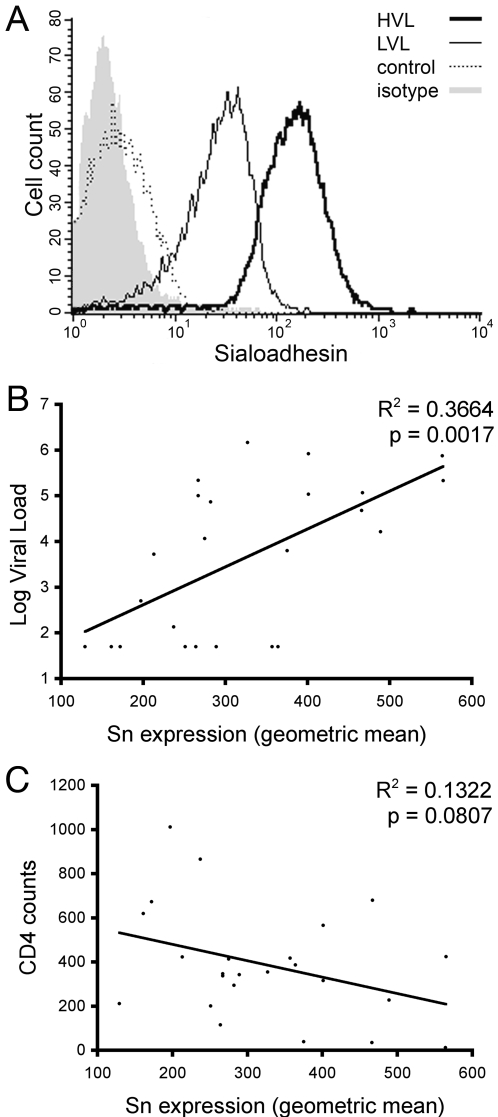
Sn expression on CD14^+^ monocytes from HIV seropositive individuals. (A) Representative frequency histograms of relative Sn expression on CD14^+^ monocytes isolated from subjects with high viral load (HVL, 214,000 RNA copies/ml, thick black line), low viral load (LVL, 6,350 RNA copies/ml, thin black line) and a seronegative control (dotted line). The isotype-matched control mAb is shown in the shaded profile. (B) Correlation analysis of Sn expression and viral load. Sn expression on CD14^+^ monocytes from HIV seropositive subjects (Table 1, n = 24) was determined by flow cytometry and quantified as a geometric mean for each subject. Pearson's correlation analysis showed statistical significance between Sn expression and the log of the subject's viral load (p<0.0017). (C) Correlation analysis of Sn expression and CD4 (counts/ml) revealed no significant relationship (p<0.08).

We previously reported elevated Sn gene expression on CD14^+^ monocytes from subjects infected with HIV-1 [11]. To determine if Sn was differentially expressed on peripheral monocytes, immunomagnetically sorted CD14^+^ monocytes from HIV-1 seropositive subjects (n = 24) and HIV-1 seronegative controls (n = 10) were analyzed by flow cytometry. Sialoadhesin expression, quantified as the geometric mean, along with the subject's viral load (RNA copies/ml), CD4 count (cells/ml) and therapeutic status (on or off HAART) are shown in Table 1. The range of Sn expression on CD14^+^ monocytes from subjects infected with HIV-1 and seronegative controls is depicted in representative frequency histogram plots (Figure 1A). To determine the relationship between Sn expression and viral load, and CD4 count, HIV-1 seropositive subjects (n = 24) were evaluated using Pearson's correlation analysis. Correlation of Sn expression was statistically significant for viral load (p<0.0017) (Fig. 1B) but not with CD4 count (p<0.08) (Fig. 1C). In a follow-up study, three seropositive subjects, initially with detectable viral loads and high Sn expression, were subsequently retested for Sn expression after successful HAART treatment suppressed viral replication to <50 copies/ml. In all three cases, Sn dropped to <200 (geometric mean) reinforcing the link between Sn expression and viral load (data not shown).

**Table 1:** Clinical data and Sn expression levels from controls and HIV-1 seropositive subjects.

Subject	Sn^a^ Expression	Viral Load^b^×10^3^	CD4^c^	Therapy^d^
C1	129			
C2	229			
C3	218			
C4	72			
C5	120			
C6	318			
C7	138			
C8	194			
C9	193			
C10	295			
V58	357	<0.05	417	+
V60	264	<0.05	115	+
V67	364	<0.05	386	+
V68	251	<0.05	201	+
V77	289	<0.05	343	+
V91	161	<0.05	619	+
V92	172	<0.05	673	+
V94	129	<0.05	211	+
V87	237	0.14	865	+
V72	197	0.50	1011	+
V69	327	1.47	354	+
V73	213	5.26	423	+
V61	375	6.35	39	-
V90	275	11.60	414	-/STI
V78	489	16.40	227	-/STI
V70	466	47.50	35	+
V59	282	73.00	294	+
V95	267	100.00	337	+
V71	401	108.00	316	-/STI
V53	467	116.00	679	-/STI
V76	565	214.00	424	-
V54	267	219.00	347	+
V75	564	750.00	12	+
V74	401	833.00	566	-

Data for controls (C, n = 10), HIV-1 seropositive subjects (V, n = 24)

Data sorted on increasing viral load

a Sialoadhesin (Sn) expression on CD14+ monocytes by flow cytometry quantified as the geometric mean

b HIV RNA copies/ml

c CD4 positive cells/ml

d On (+) or off (−) highly active antiretroviral therapy; Structured treatment interruption (STI)

### Interferons induce Sn expression *in vitro*

**Figure 2:**
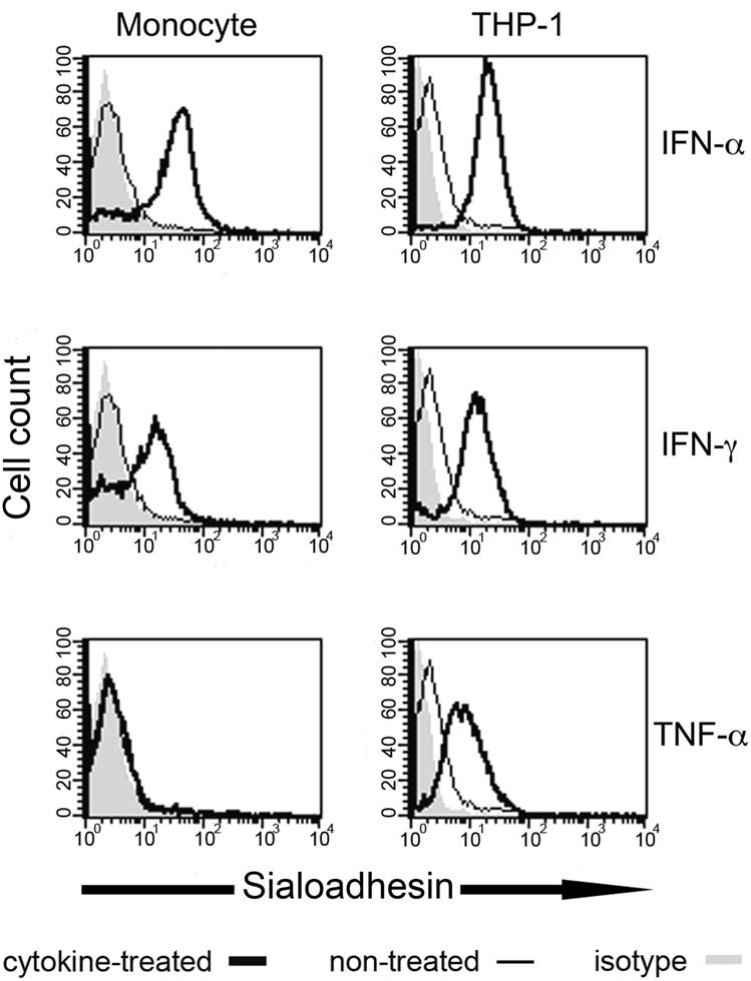
Interferon-α and -γ induce Sn expression on CD14^+^ monocytes and THP-1 cells. Cells were cultured in 500 U/ml IFN-α, 100 U/ml IFN-γ or 10 ng/ml TNF-α at 37°C for 48 h and analyzed for Sn expression by flow cytometry. Sn expression on IFN-α-, IFN -γ- or TNF-α-treated cells (thick black lines) and untreated cells (thin black line) were relative to an isotype-matched mAb control (shaded region). Results shown are representative histograms from three independent experiments using monocytes from three seronegative donors.

We wanted to know what soluble factors might drive monocyte Sn expression in the periphery. In mice, Sn expression is associated with inflammation in a subset of macrophages [13]; an analogous Sn response was elicited from human monocyte-derived macrophages when treated with a combination of tumor necrosis factor (TNF)-α and IFN-γ [13]. However, in individuals infected with HIV-1, viremia coincided with induction of type I IFN-stimulated gene transcripts and not proinflammatory cytokines [20]. We investigated if TNF-α, IFN-γ or IFN-α, would effectively induce Sn expression on CD14^+^ monocytes from HIV-1 seronegative subjects. To prevent attachment activation, freshly isolated CD14^+^ monocytes were cultured in low-adherent well plates and assayed for Sn expression by flow cytometry within 48 h. We found that Sn expression was induced by both IFN-α and IFN-γ but not TNF-α (Fig. 2). When analyzed for their effect on THP-1 cells, a monocytic cell line, IFN-α, IFN-γ and TNF-α all induced Sn expression indicating that Sn is differentially controlled in monocytes and THP-1 cells (Fig. 2). We also tested the hypothesis that HIV-1 might directly induce Sn expression on monocytes. When either PBMC or monocytes were treated with 5 ng/ml HIV-1_NL4-3_, flow cytometry analysis 48 h later did not detect an increase in Sn expression (data not shown). While not definitive, our data suggests that Sn expression in subjects infected with HIV-1 is orchestrated by IFN, which is an innate immune response to viremia.

### Sn expression in a transduced THP-1 cell line

**Figure 3:**
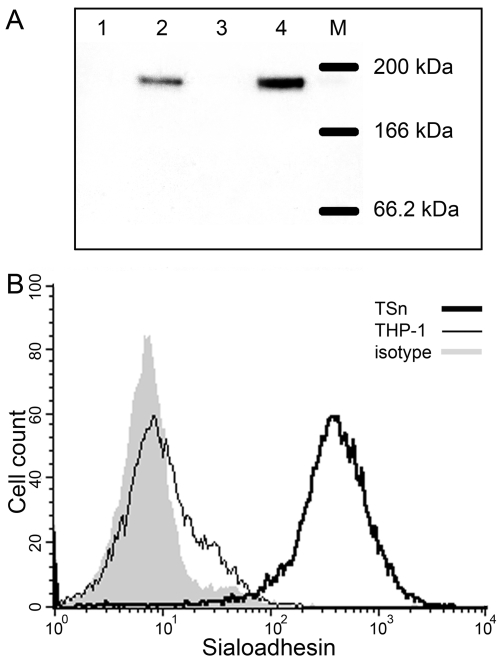
Constitutive Sn expression in THP-1 by gene transduction (TSn). (A) Immunoblot analysis of Sn protein expression. THP-1 cell line was transduced with a plasmid encoding Sn cloned downstream of the high-level constitutive promoter CMV. Cell lysates were standardized, reduced with DTT and 10 µg of protein were loaded into each well: monocytes (lane 1), IFN-α-induced monocytes (lane2), THP-1 (lane 3) and TSn (lane 4) (M, molecular size marker). (B) Histogram of relative distribution of Sn on the cell surface of TSn clone. TSn (thick black line) and THP-1 cells (thin black line) were evaluated for Sn expression by flow cytometry using anti-Sn mAb 7D2 relative to the background isotype-matched control mAb (shaded region).

Sialoadhesin was cloned by PCR from monocyte RNA obtained from an HIV-1 seropositive individual with a high viral load. The cloned structural gene (5130 base pairs) was sequenced and compared with the *SIGLEC 1* in the National Center for Biotechnology Information (NCBI) database (accession number NM_023068). Sequence data from the cloned *SIGLEC 1* consistent with the NCBI sequence with the exception of two single nucleotide polymorphisms (dbSNP: 6037651 and dbSNP: 709012) in the cloned gene, which generated two sense mutations outside the sialic acid-binding domain. Neither polymorphism is known to influence the expression or alter the function of Sn. Subsequently, *SIGLEC 1* was subcloned into an expression cassette with a CMV promoter for constitutive expression and then packaged into a lentiviral vector to transduce monocytic THP-1 cells. An Sn-expressing cell line, TSn, was generated by clonal expansion of a single transductant. Analyses of the TSn cell line showed the 190 kDa Sn protein by Western blot (Figure 3A) and flow cytometry (Figure 3B).

### Sn-dependent binding of HIV-1

**Figure 4:**
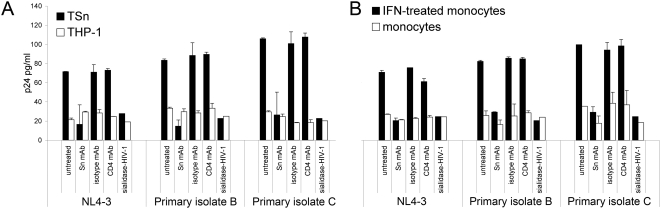
Sn binds HIV-1 *in vitro*. (A) TSn binds HIV-1 in an Sn-dependant manner. TSn and THP-1 were incubated with lab-adapted HIV-1_NL4-3_, an HIV-1 clade B primary isolate or clade C primary isolate for 1 h at 37°C, washed and then assayed for HIV-1 p24 by ELISA. HIV-1 binding to Sn was abrogated by pretreatment of cells with Sn mAb 7D2 or by pretreatment of HIV-1 with broad-spectrum sialidase. Pretreatment with IgG1 isotype control or CD4 mAbs did not reduce binding. Data presented are the average of 3 separate experiments. (B) IFN-α-treated CD14^+^ monocytes bind HIV-1 in an Sn-dependant manner. HIV-1 binding assays were also performed on CD14^+^ monocytes from seronegative controls treated with 500 U/ml IFN-α to induce Sn expression. Pretreatment with Sn mAb 7D2 and sialidase dramatically reduced HIV-1 binding while pretreatment with IgG1 isotype control or CD4 mAbs had little effect. Data represents monocytes from four separate donors. Error bars represent SD.

Since Sn is capable of binding sialic acid conjugates on adjoining cells, we considered the possibility that Sn would effectively bind HIV-1 via the sialic acid residues on gp120. In binding assays, TSn cells (1×10^6^ cells/ml) were incubated with 8 ng/ml HIV-1_NL4-3_ for 1 h at 37°C. After extensive washing to remove nonspecifically bound virus, TSn cells were lysed and assayed for p24 by ELISA. When compared to THP-1 controls, TSn cells bound approximately four-fold more HIV-1_NL4-3_ (Fig. 4A). To characterize the TSn-HIV-1 interaction, TSn cells were preincubated with an anti-Sn monoclonal antibody (mAb) 7D2 [13], which recognizes the V-set, N-terminal sialic acid binding region of Sn. Preincubation with mAb 7D2 abrogated HIV-1_NL4-3_ binding to TSn demonstrating that Sn is required for HIV binding. As a control, pretreatment with a IgG1 isotype antibody did not interfere with virus binding (Figure 4A). To determine whether the HIV receptor CD4 was contributing to HIV binding in this assay, THP-1 and TSn cells were preincubated with an anti-human CD4 mAb prior to challenge with HIV-1_NL4-3_. There was no change in HIV-1_NL4-3_ binding for either THP-1 or TSn respectively, compared to the untreated control (Figure 4A). With solid evidence that Sn was responsible for HIV-1_NL4-3_ binding to TSn, HIV-1_NL4-3_ was pretreated with sialidase to remove terminal sialic acids. Sialidase-treated HIV-1_NL4-3_ resulted in significantly reduced binding to TSn cells but had no appreciable effect on THP-1 cells (Fig. 3A). These findings demonstrated that HIV-1_NL4-3_ binding to TSn is dependent on a viral sialic acid ligand and cellular expressed Sn.

During infection, the hyper-mutation rate of HIV generates significant genetic variation in circulating virus. To analyze whether genetic diversity might impact virus binding, HIV-1 primary isolates from clade B and clade C were evaluated for their ability to bind Sn. Results showed that primary isolates bound to TSn cells in a manner similar to HIV-1_NL4-3_ and that binding was abrogated by pretreatment with the anti-Sn mAb 7D2 or virus pretreated with sialidase (Fig. 4A).

Next, we examined the capacity of Sn expressed on human monocytes to bind HIV-1. Sn expression was induced by treating CD14^+^ monocytes from HIV seronegative controls with 500 U/ml IFN-α for 48 h. IFN-α-induced monocytes incubated with HIV-1_NL4-3_ bound approximately three-fold more virus compared to non-induced monocyte controls (Fig. 4B). Preincubation with mAb 7D2 significantly reduced HIV-1_NL4-3_ binding to IFN-α-treated monocytes comparable to that observed for non-induced monocytes, indicating that IFN-α-induced HIV-1_NL4-3_ binding was due to Sn alone. When HIV-1_NL4-3_ was pretreated with sialidase, subsequent binding to Sn-expressing monocytes was impaired (Fig. 4B). Additional binding assays using primary isolates with IFN-α-treated monocytes demonstrated that Sn bound primary isolates with equal effectiveness. As before, virus adsorption could be prevented to a significant extent by pretreatments with anti-Sn mAb 7D2 or sialidase.

### Sn facilitates *trans* infectivity

**Figure 5:**
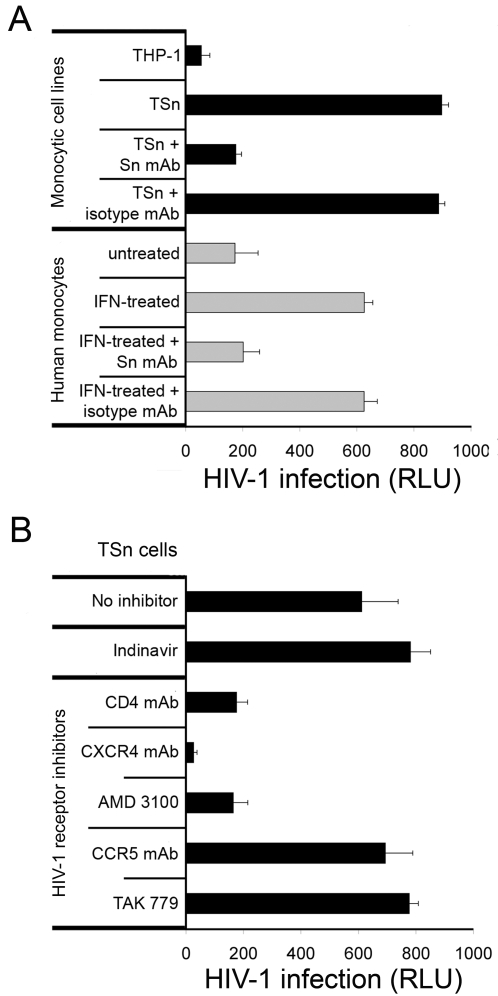
Sn-dependent *trans* infection of reporter cells TZM-bl. (A) Sn-expressing cells, TSn and IFN-α-induced monocytes, bind HIV-1 and infect TZM-bl cells in *trans*. Cells were incubated with HIV-1_NL4-3_ for 1 h, washed and then cocultured with TZM-bl cells for 48 h. HIV-1 infection of TZM-bl was defined by luciferase expression and quantified as relative light units (RLU). Cells pretreated with Sn mAb 7D2 showed significantly reduced capacity to facilitate *trans* infection of TZM-bl cells while mAb IgG1 isotype control had no effect. (B) HIV-1 receptor inhibitors block *trans* infection. Receptor and coreceptor requirements for *trans* infection of TZM-bl cells were tested by incubating TZM-bl cells with receptor inhibitors including mAbs to CD4, CXCR4 or CCR5, and small molecules AMD3100 or TAK779 prior to adding TSn with bound HIV-1_NL4-3_. The CD4, CXCR4, CCR5, AMD3100 and TAK779 receptor inhibitors were tested individually with TZM-bl and did not induce luciferase expression (data not shown). As a control, productive infection of TSn cells was prevented by addition of indinavir (100 µM). Data presented are the average of three separate experiments. Error bars represent SD.

Having shown that Sn avidly binds HIV-1, we explored the possibility that this might impact virus infectivity. To test this theory, we employed an infectious lab-adapted HIV-1_NL4-3_ strain and a reporter cell line TZM-bl, which expresses luciferase when infected [21]. TZM-bl cells, which are permissive to HIV-1_NL4-3_ infection, express the viral receptor CD4 and coreceptors CCR5 and CXCR4. When THP-1 cells were pulsed with concentrated HIV-1_NL4-3_ stock for 1 h, washed extensively to remove nonspecifically bound virus and cocultured for 48 h with adherent TZM-bl cells, luciferase activity was slightly above background at 55 relative light units (RLU) (Fig. 5A). In contrast, TZM-bl cells cocultivated with HIV-1_NL4-3_-pulsed TSn cells, expressed high levels of luciferase (897 RLU) indicating robust *trans* infection by HIV-1_NL4-3_. To verify that *trans* infection was dependent on Sn binding of viral sialic acid conjugates, TSn was preincubated with mAb 7D2 prior to HIV-1_NL4-3_ exposure. Consistent with the Sn- HIV-1_NL4-3_ binding assay, pre-treatment of TSn with mAb 7D2 reduced infection in the reporter cells as illustrated by the dramatic reduction in luciferase activity (Fig. 5A).

We then tested the potential of human CD14^+^ monocytes to facilitate *trans* infection of TZM-bl cells by isolating monocytes from HIV-1 seronegative subjects and inducing Sn expression with a 48 h pre-treatment of IFN-α. When Sn-expressing human monocytes were pulsed with HIV-1_NL4-3_ and subsequently cocultured with TZM-bl cells, luciferase activity exceeded 625 RLU indicating Sn-expressing human monocytes were capable of facilitating *trans* infection (Fig. 5A). By comparison, non-induced monocytes, which have minimal Sn expression (Fig. 2), exhibited low luciferase activity. As a control, IFN-α treatment of TZM-bl cells resulted in luciferase activity equal to that of background (data not shown). These results suggest that peripheral monocytes expressing Sn are capable of binding HIV-1 and presenting infectious virus to susceptible cells.

It was imperative that Sn-bound HIV-1_NL4-3_ be the only source of virus in these *trans* infection assays. We considered the possibility that HIV-1_NL4-3_-infected TSn cells would provide a secondary source of virus. To block any potential release of infectious virus from TSn cells during the 48 h culture period, assays were performed using indinavir (100 µM), a protease inhibitor. When indinavir was added to the cultures, there was no decrease in luciferase activity effectively demonstrating that TZM-bl cells were infected by *trans* infection of Sn-bound HIV-1_NL4-3_ and not from infected TSn cells (Fig. 5B).

The fact the Sn binds HIV-1_NL4-3_ and facilitates *trans* infection raised the possibility that normal receptor CD4 and chemokine coreceptor requirements might be altered by Sn-bound HIV-1_NL4-3_. We tested this possibility by treating TZM-bl cells with blocking antibodies and small molecules that prevent HIV infection by binding to CD4 and coreceptors CXCR4 and CCR5. Pretreatment of the TZM-bl cells with anti-CD4 mAb B4 [22] reduced subsequent *trans* infection to background levels indicating that CD4 remains essential for infection in this model (Fig. 5B). Blocking coreceptor CXCR4 with either a small molecule, AMD3100 [23] or an anti-CXCR4 mAb 12G5 [24] also abrogated *trans* infection of the TZM-bl cells (Fig. 5B). Also, two inhibitors of the CCR5 coreceptor, an anti-CCR5 mAb 2D7 [25]] and TAK779 [26]], which should not interfere with *trans* infection since HIV-1_NL4-3_ uses the CXCR4 coreceptor, had no effect. From these data we conclude that Sn does not bypass receptor and coreceptor requirements for the T-tropic (X4) HIV-1_NL4-3_ strain.

### HIV-1 infectivity enhanced by Sn

**Figure 6:**
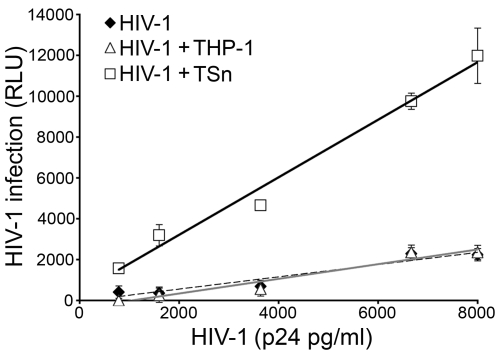
Sn-expressing cells capture HIV-1_NL4-3_ and enhance infectivity. TZM-bl cell cultures were seeded with various concentrations of HIV-1_NL4-3_ (800–8000 pg/ml). Monocytic cells, TSn or THP-1 cells, were added and cocultured for 48 h. The capacity of Sn to capture HIV-1_NL4-3_ in the cell culture medium and *trans* infect TZM-bl cells was analyzed for TSn, THP-1 and cell-free virus. Luciferase expression in HIV-1_NL4-3_-infected TZM-bl cells was quantified as relative light units (RLU). Results were compiled from 3 separate experiments. Error bars represent SD.

Since individuals with detectable viral loads have circulating cell-free HIV-1, we examined the capacity of Sn-expressing cells to adsorb cell-free virus in conditioned medium and then *trans* infect reporter cells. In a capture assay, TZM-bl cell cultures were inoculated with HIV-1_NL4-3_ followed by subsequent addition of either TSn or THP-1 cells. The baseline value for infectivity was established with cell-free virus in the absence of any monocytic cells. After a 48 h incubation, increasing concentrations of cell-free virus correlated with luciferase expression and therefore infection of TZM-bl cells (Fig. 6). Addition of THP-1 cells had minimal impact on TZM-bl infection and was comparable to cell-free virus. However, when TSn cells were added, infectivity of the reporter cells increased dramatically as demonstrated by elevated luciferase activity (Fig. 6). In fact, over the range of HIV-1_NL4-3_ concentrations from 800 pg/ml to 8000 pg/ml, virus infectivity was enhanced over 5-fold in the presence of Sn compared to cell-free virus. These data indicate that Sn captures cell-free virus and increases the rate of infection suggesting that Sn may function as an enhancer of HIV infection in the periphery.

## Discussion

Myeloid lineage cells including monocytes, macrophages and dendritic cells, tightly regulate Sn expression in response to specific disease conditions. Presently, the specific factor(s) elevating Sn during HIV-1 infection are not known. We show that Sn expression correlates with viral loads that range from undetectable (<50 copies/ml) to unsuppressed (>800,000 RNA copies/ml). This finding was consistent with our previous microarray study [11] which suggested that viral load was a determining factor in Sn expression. This interpretation was further supported by data from subjects whose viral loads, initially high, were suppressed to undetectable levels following HAART treatment and exhibited a concurrent decrease in Sn expression. In combination with our *in vitro* data showing that IFN-α and IFN-γ induce Sn expression in cultured human monocytes and THP-1 cells, it is possible that either of these cytokines drives Sn expression during HIV-1 infection. IFN-γ, which is associated with immune activation, is produced by T cells and NK cells during the acute period of infection [27]. By comparison, IFN-α, which is present in the serum of individuals infected with HIV-1 [28], [29], is released by plasmacytoid dendritic cells in an innate antiviral response [30], [31]. In a study by Tilton et al., monocyte production of proinflammatory cytokines, IL-1β, IL-6 and TNF-α was diminished in HIV viremia suggesting that monocyte function was impaired during unsuppressed viral replication [20]. Coincident with the loss of monocyte function was an increase in Sn expression. Importantly, this particular phenotype could be recapitulated by stimulating monocytes with IFN-α [20]. Further substantiating the link between Sn expression and viral load was an observation in a treatment naïve population that Sn mRNA expression in CD14+ monocytes dramatically increased shortly after HIV-1 infection and continued to rise in patients who progressed to AIDS [16]. Since high viral load is a common feature of AIDS, elevated Sn expression would be predicted in these patients. In aggregate, our data suggest that elevated Sn expression on monocytes is consistent with an antiviral response, as opposed to a restricted marker of inflammation observed in tissue macrophages associated with rheumatoid arthritis [13]. While our findings indicate that either IFN-α or IFN-γ could induce Sn expression in peripheral monocytes, determining the specific conditions and cytokines responsible for Sn induction will require additional research.

Our findings present the first characterization of a monocyte protein that adsorbs HIV-1 and facilitates *trans* infection. A similar capability to bind HIV-1 and effect *trans* infection has been described for other proteins including syndecan and DC-SIGN. Syndecan, a cell surface heparan sulfate proteoglycan expressed on macrophages, endothelial cells and fibroblasts, facilitates *trans* infection of T cells and can preserve virus infectivity for an extended period [32], [33]. When expressed on macrophages, syndecan functions as a *cis*-oriented HIV-1 receptor with the virus exploiting similar attachment motifs on both syndecan and CCR5 [34]. *Trans* infection has also been demonstrated in DCs, which scavenge and internalize pathogens in tissues and then migrate to lymph nodes where they present antigens to resting T cells [35]. A subset of DCs express DC-SIGN (dendritic cell – specific intercellular adhesion molecule-grabbing nonintegrin), a type II transmembrane protein with a C-type lectin ectodomain that can adsorb HIV-1 but does not serve as receptor for viral fusion. Initially, DC-SIGN-dependent *trans* infection of permissive cells was shown to occur in the absence of infection or even internalization of the virus [36]. However, recent reports present alternative pathways by which DC-SIGN may support infection of permissive cells. DC-SIGN has been shown to rapidly internalize intact HIV-1 into non-degradative compartments where the virus remains competent to infect permissive cells [37]. Moreover, it has been shown that DC-SIGN-mediated internalization is not required for *trans*-enhancement but instead DC-SIGN facilitates productive *cis*-infection of immature DCs and subsequent infection of T cells [38]. Also it has been reported that DC-SIGN is not required for DC-mediated *trans* infection based on observations that neither down-regulating DC-SIGN expression nor binding of an anti-DC-SIGN antibody adversely impacted DC-mediated *trans* infection [39]. By comparison, our experiments conclusively show that Sn-dependent *trans* infection is not due to productive infection of TSn cells since *trans* infection assays were done in the presence of indinavir, a potent HIV protease inhibitor. Furthermore, our results are consistent with Sn being solely responsible for *trans* infection since the anti-Sn mAb 7D2, which recognizes the Sn sialic acid binding region, effectively abrogates *trans* infection of the reporter cells.

A possible mechanism that would explain Sn-enhanced infectivity would be that by binding both virus and target cells, Sn effectively increases the localized virus concentration bringing HIV-1 in close proximity to CD4 and chemokine coreceptors on the target cell. Early characterization of Sn expressed on macrophages identified it as a lymphocyte adhesion molecule that binds sialic acid conjugates on T cells [12], specifically CD43 [40]. Having clearly demonstrated that Sn binds HIV-1, Sn could possibly perform dual functions, with some Sn protein capturing free HIV-1 while other Sn molecules would engage sialic acid conjugates on the target cell. This interaction would overcome the electrostatic repulsive forces created by net negative charges on the virus and the target cell surface. Repulsive forces modulate virus-cell interactions as demonstrated by treating primary mononuclear cells with purified sialidase, which increased susceptibility to HIV-1 infection by reducing electrostatic repulsion [41]. The hypothesis is that desialylation enhances the interaction of viral gp120 and cell associated CD4/chemokine coreceptors thereby promoting HIV-1-mediated syncytium formation. These observations suggest that HIV-1 can exploit either condition: When sialic acid conjugates are intact, Sn binds the virus and subsequently delivers the virus to permissive cells. Or, if sialic acids are cleaved from gp120 by endogenous sialidase activity, the electrostatic repulsion is reduced permitting viral interaction with the cellular CD4/chemokine coreceptors.

In this study, we used human monocytes and a stably transduced cell line to demonstrate the capacity of Sn to bind HIV-1 and facilitate the *trans* infection of permissive cells. While the biological relevance of Sn expression on circulating monocytes remains to be determined, the potential impact of enhancing viral infectivity and possibility disseminating HIV-1 via monocyte trafficking to the CNS are clearly important considerations.

## Materials and Methods

### Cells and viruses

Monocytes for *in vitro* experiments were isolated from HIV-1 seronegative healthy donors (Blood Centers of the Pacific, San Francisco, CA). In brief, cells were flushed out of leuko-reduction filters with 40 ml PBS without Ca^2+^ and Mg^2+^ using a syringe. Monocytes were enriched by negative selection using RosetteSep according to the manufacturer (StemCell Technologies, Vancouver, BC), with >80% purity by flow cytometry. THP-1 cells were obtained from the Cell Culture Facility (UCSF, San Francisco, CA) and cultured in RPMI-1640 supplemented with 10% FBS, 1.0 µg/ml gentamicin and 2 mM L-glutamine at 37°C in 5% CO2. Lab-adapted HIV-1_NL4-3_ was a generous gift from Dr. Joe Wong. Primary isolates 92US660 (HIV-1 B1) and MW/93/959 (HIV-1 C2) and reporter cell line TZM-bl were obtained from NIH AIDS Research and Reference Reagent Program (Germantown, MD). Lab-adapted HIV-1_NL4-3_ and primary isolates HIV-1 B1 and C2 were replicated in PBMC from 3 seronegative donors (Blood Centers of the Pacific) using a standard *in vitro* HIV-1 replication protocol (SRA Life Sciences; available on the NIH AIDS Research and Reference Reagent Program website). TZM-bl cells were also obtained from the NIH AIDS Research and Reference Reagent Program and cultured in DMEM H-21 supplemented with 10% FBS and 1.0 µg/ml gentamicin at 37°C in 5% CO2.

### Quantification of *in vivo* Sn expression by flow cytometry

This study received IRB approval from the University of California, San Francisco and the Veterans Affairs Medical Center, San Francisco and written consent was obtained from participating subjects. For flow cytometric analysis, CD14^+^ monocytes were isolated within 2 h from fresh blood obtained from donors recruited at the San Francisco VA Medical Center. In brief, 30 ml of blood was collected in BD Vacutainer CPT tubes (BD, Franklin Lakes, NJ) and centrifuged to enrich for PBMC. Monocytes were isolated from PBMC by positive immunomagnetic cell sorting using CD14 Microbeads (Miltenyi Biotec, Auburn, CA) which yielded >90% purity when assayed by flow cytometry (FACSCalibur; BD Biosciences, San Jose, CA) using anti-CD14-FITC (BD Pharmingen, San Diego, CA). Sn expression was quantified by flow cytometry using anti-human Sn monoclonal antibody (mAb) 7D2 (Novus Biologicals, Littleton, CO) and secondary PE anti-mouse IgG1 (BD Pharmingen). Mouse IgG1 (BD Pharmingen) was used as a non-specific control antibody. The geometric mean of the channel value for 10^4^ cells was determined using CellQuest software (BD Biosciences).

### *In vitro* induction of Sn

THP-1 cells or CD14^+^ monocytes from HIV-1 seronegative controls (1×10^6^ cells) were stimulated with 500 U/ml IFN-α2a (PBL Biomedical Laboratories, Piscataway, NJ), 100 U/ml IFN-γ or 10 ng/ml TNF-α (R&D Systems, Minneapolis, MN) in RPMI-1640 supplemented with 10% FBS, 1.0 µg/ml gentamicin and 2 mM L-glutamine at 37°C in 5% CO_2_ for 48 h in Costar Ultra Low Attachment plates (Corning, Lowell, MA) with rotation to prevent adherence.

### SN cloning and constitutive expression in clone TSn

Total RNA was extracted from CD14^+^ monocytes isolated from an HIV-1 seropositive individual using RNeasy Mini kit (Qiagen, Valencia, CA). cDNA was generated using Superscript III First-strand cDNA Synthesis kit (Invitrogen, Carlsbad, CA). SN was cloned by RT-PCR (94°C for 2 min followed by 35 cycles of 94°C for 30 sec, 58°C for 1 min, 72°C for 6 min with extension at 72°C for 10 min) using FailSafe PCR PreMix Selection Kit (Epicentre Biotechnologies, Madison, WI). Primers were as follows: forward 5′- TGA TAT CTT AAG GCA CAA GAA CCT GCT ATG G -3′ and reverse 5′- AGA TAT CTA GAC AAC ACC ACT GGT CAG CC -3′. The PCR product was inserted into pCR2.1 and sequenced (Cleveland Genomics, Cleveland, OH). SN was cloned into pENTR1A then subcloned into pLenti6/V5-Dest using Gateway® technology and then packaged into the lentiviral vector using ViraPower Lentiviral Expression System in a 293FT cell line (all from Invitrogen). Lentiviral suspensions (3 ml) were combined with THP-1 cells (1×10^7^) and mixed with polybrene (8 µg/ml). Lentivirus was loaded onto THP-1 cells by spinoculation (600×g for 1.5 h). After, cells were resuspended in RPMI-1640 supplemented with 10% FBS, 1.0 µg/ml gentamicin and 2 mM L-glutamine and cultured at 37°C in 5% CO_2_. Clonal selection was done in the presence of 5 µg/ml blasticidin.

### Immunoblot

THP-1, TSn, monocytes and monocytes stimulated with 500 U/ml IFN-α2a (2×10^6^) were washed with PBS and lysed with ice-cold lysis buffer containing 10 mM HEPES pH 7.9, 10 mM KCl, 0.1 mM EDTA, 0.1 mM EGTA, 0.5 mM PMSF, 1 mM DTT and 0.5% NP-40. Cell lysates were normalized by protein concentration, reduced with DTT and 10 µg of sample protein were loaded into a 4–12% Bis-Tris gel (Invitrogen). Proteins were fractionated in reducing conditions by electrophoresis for 1 h at 200 V, electroblotted for 1 h at 100 V onto a polyvinylidene difluoride (PVDF) membrane (GE Healthcare, Picastaway, NJ). The PVDF membrane was probed with Sn mAb 7D2 (1∶1000; Novus Biologicals) and secondary peroxidase-labeled horse anti-mouse IgG (1∶5000; Vector Laboratories, Burlingame, CA) then visualized with ECL plus on a Typhoon Scanner (GE Healthcare, Picastaway, NJ).

### *In vitro* HIV binding assays

Cellular binding of lab-adapted HIV-1_NL4-3_ and primary isolates clade B (HIV-1 92US660) and clade C (MW/93/959) was assessed using THP-1, TSn, monocytes and IFN-α2a-stimulated monocytes. In brief, 2.5×10^5^ cells/250 µl PBS were pulsed with 2 ng p24-normalized virus stocks for 1 h at 37°C. Cells were vigorously washed 5 times with PBS by vortexing and centrifugation at 300×g. To demonstrate that increased binding of HIV-1 to TSn and monocytes stimulated with IFN-α2a involved Sn, cells were pretreated with 0.5 µg Sn mAb 7D2, mAb IgG1 isotype control (Novus) or CD4 mAb RPA-T4 (BD Bioscience) for 1 h at 37°C. Sialic acid residues on HIV-1 were removed with 4×10^−4^ U/ml sialidase from *Clostridium perfringens* (Roche, Indianapolis, IN) for 1 h at 37°C. HIV-1 bound to cells was quantified by HIV p24 ELISA (Zeptometrix, Buffalo, NY).

### *Trans* infection assay

TZM-bl cells were distributed into 96-well plates (5×10^3^ cells/well) and cultured in RPMI-1640 supplemented with 10% FBS, 1.0 µg/ml gentamicin for 48 h at 37°C in 5% CO_2_. On the same day, CD14^+^ monocytes from controls were isolated and half were stimulated with 500 U/ml IFN-α2a for 48 h at 37°C in 5% CO_2_ with rotation to prevent adherence to plastic. After, THP-1, TSn, monocytes and monocytes stimulated with IFN-α2a (1×10^6^ cells/250 µl PBS) were pulsed with 2 ng HIV-1_NL4-3_ for 1 h at 37°C, washed vigorously 5 times with PBS by vortexing and centrifugation at 300×g, then resuspended in 1 ml RPMI-1640+10% FBS. To demonstrate involvement of Sn, cells were pretreated with 0.5 µg Sn mAb 7D2 or mAb IgG1 isotype control for 1 h at 37°C and washed 3 times with PBS. To confirm which coreceptors were required for *trans* infection via TSn, TZM-bl cells were pretreated with 0.5 µg/ml anti-CD4 RPA-T4 (BD Bioscience), 0.5 µg/ml anti-CXCR4 12G5 or anti-CCR5 2D7, or 10 µM AMD3100 or TAK779 (all from NIH AIDS Research and Reference Reagent Program). After, the TZM-bl media was replaced with 100 µl of the cell suspension (1×10^5^ cells) and cultured for 48 h at 37°C in 5% CO_2_. Productive infection of TSn and TZM-bl cells was prevented by the presence of 100 µM indinavir protease inhibitor (NIH AIDS Research and Reference Reagent Program). Infection of the TZM-bl cells was quantified as relative light units (RLU) produced by luciferase expression using the Bright-Glo™ detection system (Promega, Madison, WI) and a SpectraMax M5 microplate reader (Molecular Devices, Sunnyvale, CA).

### Enhancement of HIV-1_NL4-3_ infectivity

The ability of Sn-expressing cells to capture HIV-1_NL4-3_ in solution and then infect TZM-bl cell *in trans* was compared to cell-free virus. TZM-bl cells (5×10^3^ cells/well) were distributed into 96-well plates and incubated for 48 h at 37°C in 5% CO_2_. After, the media was replaced with 50 µl of dilutions of HIV-1_NL4-3_ and 50 µl of 2×10^5^ THP-1 or TSn cells. The plate was rotated at 1000 rpm for 1 min and incubated for 48 h at 37°C. Luciferase activity was measured using the Bright-Glo™(Promega) and a SpectraMax M5 microplate reader (Molecular Devices).

## References

[1] Fogg DK, Sibon C, Miled C, Jung S, Aucouturier P (2006). A clonogenic bone marrow progenitor specific for macrophages and dendritic cells.. Science.

[2] Whitelaw DM (1972). Observations on human monocyte kinetics after pulse labeling.. Cell Tissue Kinet.

[3] Leon B, Lopez-Bravo M, Ardavin C (2005). Monocyte-derived dendritic cells.. Semin Immunol.

[4] Delamarre L, Pack M, Chang H, Mellman I, Trombetta ES (2005). Differential lysosomal proteolysis in antigen-presenting cells determines antigen fate.. Science.

[5] Trombetta ES, Ebersold M, Garrett W, Pypaert M, Mellman I (2003). Activation of lysosomal function during dendritic cell maturation.. Science.

[6] McElrath MJ, Steinman RM, Cohn ZA (1991). Latent HIV-1 infection in enriched populations of blood monocytes and T cells from seropositive patients.. J Clin Invest.

[7] Sonza S, Maerz A, Deacon N, Meanger J, Mills J (1996). Human immunodeficiency virus type 1 replication is blocked prior to reverse transcription and integration in freshly isolated peripheral blood monocytes.. J Virol.

[8] Fischer-Smith T, Croul S, Sverstiuk AE, Capini C, L'Heureux D (2001). CNS invasion by CD14+/CD16+ peripheral blood-derived monocytes in HIV dementia: perivascular accumulation and reservoir of HIV infection.. J Neurovirol.

[9] Liu Y, Tang XP, McArthur JC, Scott J, Gartner S (2000). Analysis of human immunodeficiency virus type 1 gp160 sequences from a patient with HIV dementia: evidence for monocyte trafficking into brain.. J Neurovirol.

[10] Williams K, Westmoreland S, Greco J, Ratai E, Lentz M (2005). Magnetic resonance spectroscopy reveals that activated monocytes contribute to neuronal injury in SIV neuroAIDS.. J Clin Invest.

[11] Pulliam L, Sun B, Rempel H (2004). Invasive chronic inflammatory monocyte phenotype in subjects with high HIV-1 viral load.. J Neuroimmunol.

[12] van den Berg TK, Breve JJ, Damoiseaux JG, Dopp EA, Kelm S (1992). Sialoadhesin on macrophages: its identification as a lymphocyte adhesion molecule.. J Exp Med.

[13] Hartnell A, Steel J, Turley H, Jones M, Jackson DG (2001). Characterization of human sialoadhesin, a sialic acid binding receptor expressed by resident and inflammatory macrophage populations.. Blood.

[14] Kirchberger S, Majdic O, Steinberger P, Bluml S, Pfistershammer K (2005). Human rhinoviruses inhibit the accessory function of dendritic cells by inducing sialoadhesin and B7-H1 expression.. J Immunol.

[15] Vanderheijden N, Delputte PL, Favoreel HW, Vandekerckhove J, Van Damme J (2003). Involvement of sialoadhesin in entry of porcine reproductive and respiratory syndrome virus into porcine alveolar macrophages.. J Virol.

[16] van der Kuyl AC, van den Burg R, Zorgdrager F, Groot F, Berkhout B (2007). Sialoadhesin (CD169) Expression in CD14+ Cells Is Upregulated Early after HIV-1 Infection and Increases during Disease Progression.. PLoS ONE.

[17] Crocker PR, Clark EA, Filbin M, Gordon S, Jones Y (1998). Siglecs: a family of sialic-acid binding lectins.. Glycobiology.

[18] Crocker PR, Kelm S, Dubois C, Martin B, McWilliam AS (1991). Purification and properties of sialoadhesin, a sialic acid-binding receptor of murine tissue macrophages.. Embo J.

[19] Munday J, Floyd H, Crocker PR (1999). Sialic acid binding receptors (siglecs) expressed by macrophages.. J Leukoc Biol.

[20] Tilton JC, Johnson AJ, Luskin MR, Manion MM, Yang J (2006). Diminished production of monocyte proinflammatory cytokines during human immunodeficiency virus viremia is mediated by type I interferons.. J Virol.

[21] Wei X, Decker JM, Liu H, Zhang Z, Arani RB (2002). Emergence of resistant human immunodeficiency virus type 1 in patients receiving fusion inhibitor (T-20) monotherapy.. Antimicrob Agents Chemother.

[22] Wang CY, Sawyer LS, Murthy KK, Fang X, Walfield AM (1999). Postexposure immunoprophylaxis of primary isolates by an antibody to HIV receptor complex.. Proc Natl Acad Sci U S A.

[23] Hendrix CW, Flexner C, MacFarland RT, Giandomenico C, Fuchs EJ (2000). Pharmacokinetics and safety of AMD-3100, a novel antagonist of the CXCR-4 chemokine receptor, in human volunteers.. Antimicrob Agents Chemother.

[24] Endres MJ, Clapham PR, Marsh M, Ahuja M, Turner JD (1996). CD4-independent infection by HIV-2 is mediated by fusin/CXCR4.. Cell.

[25] Wu L, Paxton WA, Kassam N, Ruffing N, Rottman JB (1997). CCR5 levels and expression pattern correlate with infectability by macrophage-tropic HIV-1, in vitro.. J Exp Med.

[26] Baba M, Nishimura O, Kanzaki N, Okamoto M, Sawada H (1999). A small-molecule, nonpeptide CCR5 antagonist with highly potent and selective anti-HIV-1 activity.. Proc Natl Acad Sci U S A.

[27] Sinicco A, Biglino A, Sciandra M, Forno B, Pollono AM (1993). Cytokine network and acute primary HIV-1 infection.. Aids.

[28] Stylianou E, Aukrust P, Bendtzen K, Muller F, Froland SS (2000). Interferons and interferon (IFN)-inducible protein 10 during highly active anti-retroviral therapy (HAART)-possible immunosuppressive role of IFN-alpha in HIV infection.. Clin Exp Immunol.

[29] von Sydow M, Sonnerborg A, Gaines H, Strannegard O (1991). Interferon-alpha and tumor necrosis factor-alpha in serum of patients in various stages of HIV-1 infection.. AIDS Res Hum Retroviruses.

[30] Fitzgerald-Bocarsly P (1993). Human natural interferon-alpha producing cells.. Pharmacol Ther.

[31] Schmidt B, Ashlock BM, Foster H, Fujimura SH, Levy JA (2005). HIV-infected cells are major inducers of plasmacytoid dendritic cell interferon production, maturation, and migration.. Virology.

[32] Saphire AC, Bobardt MD, Zhang Z, David G, Gallay PA (2001). Syndecans serve as attachment receptors for human immunodeficiency virus type 1 on macrophages.. J Virol.

[33] Bobardt MD, Saphire AC, Hung HC, Yu X, Van der Schueren B (2003). Syndecan captures, protects, and transmits HIV to T lymphocytes.. Immunity.

[34] fude Parseval A, Bobardt MD, Chatterji A, Chatterji U, Elder JH (2005). A highly conserved arginine in gp120 governs HIV-1 binding to both syndecans and CCR5 via sulfated motifs.. J Biol Chem.

[35] Mellman I, Steinman RM (2001). Dendritic cells: specialized and regulated antigen processing machines.. Cell.

[36] Geijtenbeek TB, Kwon DS, Torensma R, van Vliet SJ, van Duijnhoven GC (2000). DC-SIGN, a dendritic cell-specific HIV-1-binding protein that enhances trans-infection of T cells.. Cell.

[37] Kwon DS, Gregorio G, Bitton N, Hendrickson WA, Littman DR (2002). DC-SIGN-mediated internalization of HIV is required for trans-enhancement of T cell infection.. Immunity.

[38] Burleigh L, Lozach PY, Schiffer C, Staropoli I, Pezo V (2006). Infection of dendritic cells (DCs), not DC-SIGN-mediated internalization of human immunodeficiency virus, is required for long-term transfer of virus to T cells.. J Virol.

[39] Boggiano C, Manel N, Littman DR (2007). Dendritic cell-mediated trans-enhancement of human immunodeficiency virus type 1 infectivity is independent of DC-SIGN.. J Virol.

[40] van den Berg TK, Nath D, Ziltener HJ, Vestweber D, Fukuda M (2001). Cutting edge: CD43 functions as a T cell counterreceptor for the macrophage adhesion receptor sialoadhesin (Siglec-1).. J Immunol.

[41] Sun J, Barbeau B, Sato S, Tremblay MJ (2001). Neuraminidase from a bacterial source enhances both HIV-1-mediated syncytium formation and the virus binding/entry process.. Virology.

